# Supplementary figures and images for: Intracellular dynamics of the Sigma-1 receptor observed with super-resolution imaging microscopy
Source: PLoS One. 2022 May 18;17(5):e0268563. doi: 10.1371/journal.pone.0268563 (PMC9116656; doi:10.1371/journal.pone.0268563)

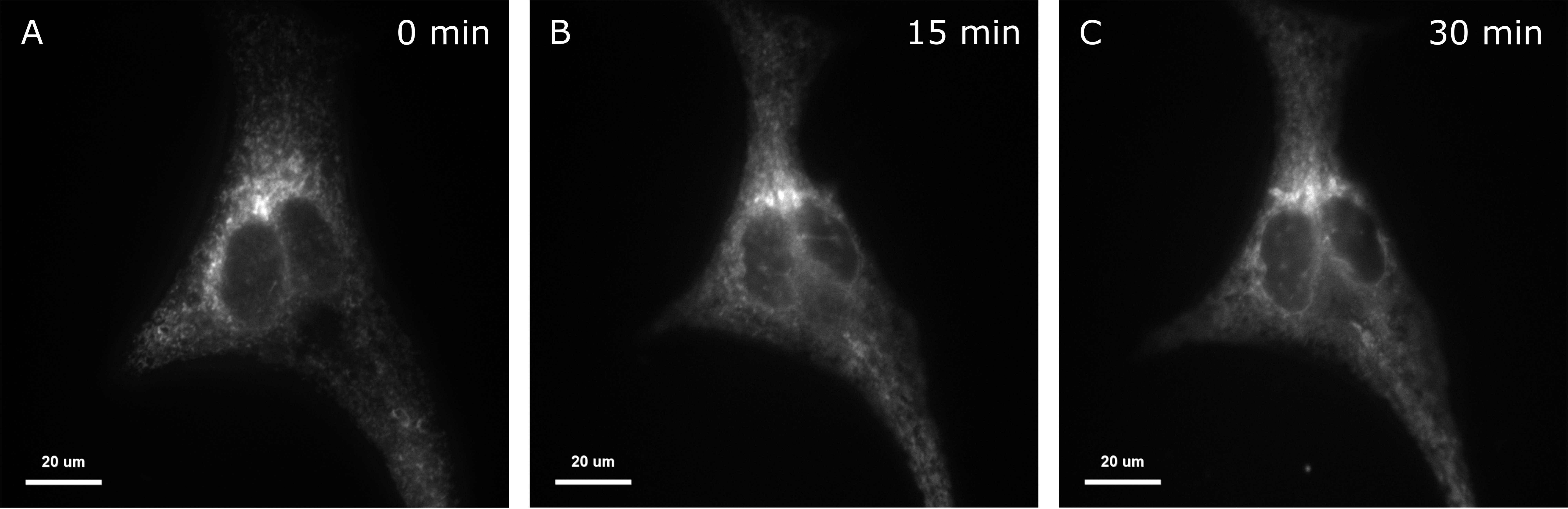

Supplement: S1 Fig — SK-OV-3 cells in 8-well glass-bottom imaging chambers were transfected with recombinant BacMam baculoviruses of Sig1R-YFP (MOI = 3) 24h before the imaging. The imaging was performed on the microscope stage heated at 37°C using 60× water (NA 1.2) objective lenses (Olympus Corp., Tokyo, Japan) and 515 nm laser for excitation. The images were taken before and 15 min and 30 min after the application of a Sig1R specific agonist PRE-084 (10 μM, final concentration) are presented as panels A, B, and C, respectively. Scale bar 20 μm. (TIF) [file pone.0268563.s001.tif]

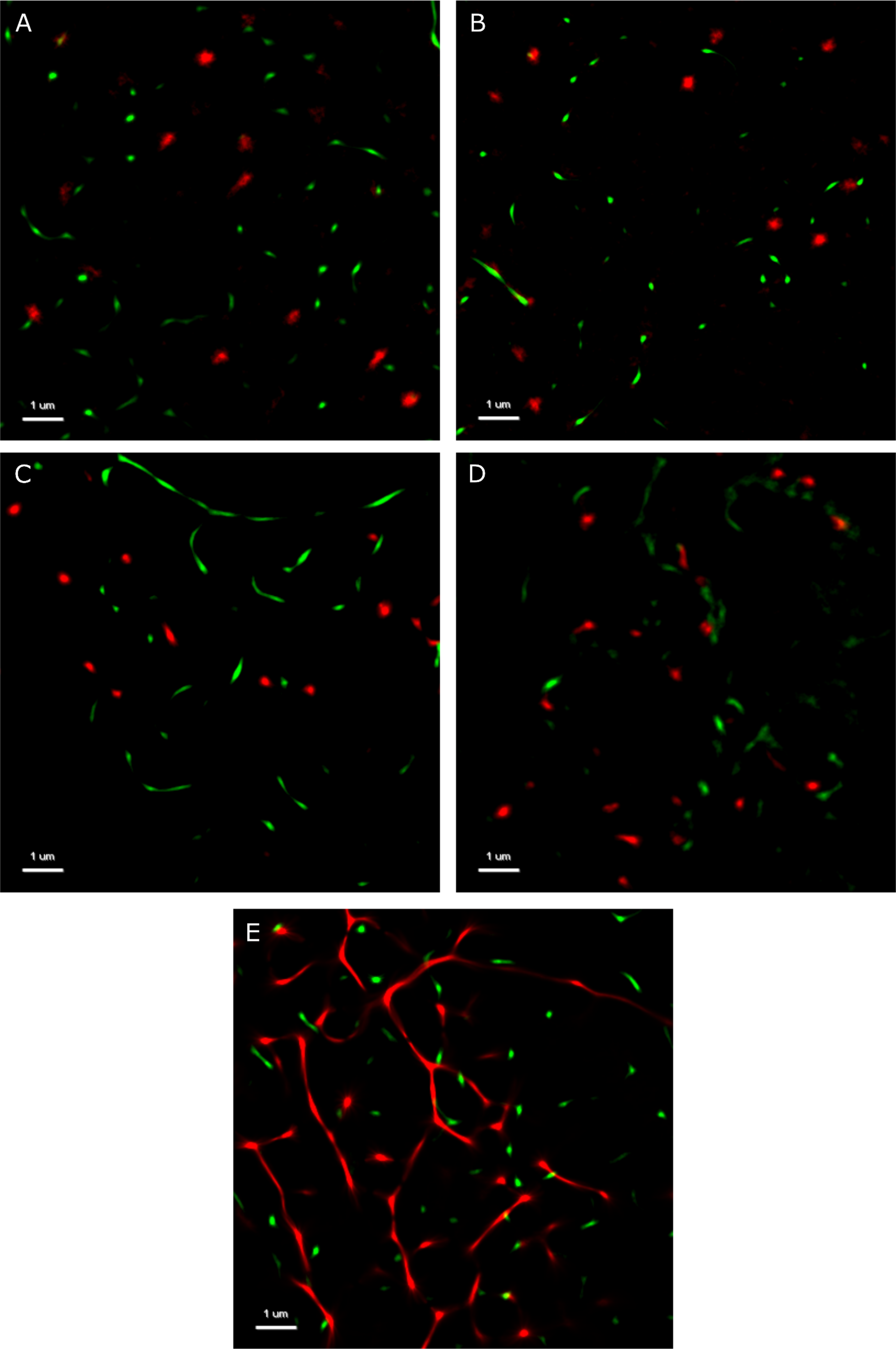

Supplement: S2 Fig — Sig1R-YFP (green) together with markers of endosomes (mCherry-Endo-14, red, (A)), lysosomes (mCherry-lysosomes-20, red, (B)), caveolae (Cav1-mRed, red, (C)), peroxisomes (mCherry-Peroxisomes-2, red (D)) and mitochondria (pmKate2-mito, red, (E)) in SK-OV-3 cells. Cells in 8-well glass-bottom imaging chambers were fixed with PFA and GA as described in the Materials and methods. Imaging of the expression of Sig1R-YFP and peroxisomes was performed in live-cells. Multichannel frames were acquired in time-laps mode (100 frames), under HILO illumination with an exposure time of 100 ms and sequential switching between 488 nm and 561 nm lasers with 60× oil (NA 1.49) objective lenses (Olympus Corp., Tokyo, Japan). Scale bar = 1 μm. The Pearson’s correlation coefficients with Sig1R-YFP were for endosomes 0.14 (A), lysosomes 0.15 (B), caveolae 0.05 (C), peroxisomes 0.03 (D) and mitochondria 0.05 (E), while for ER in the same super-resolution mode 0.26 (Fig 3F), but in diffraction-limited mode 0.79 (S4F Fig). (TIF) [file pone.0268563.s002.tif]

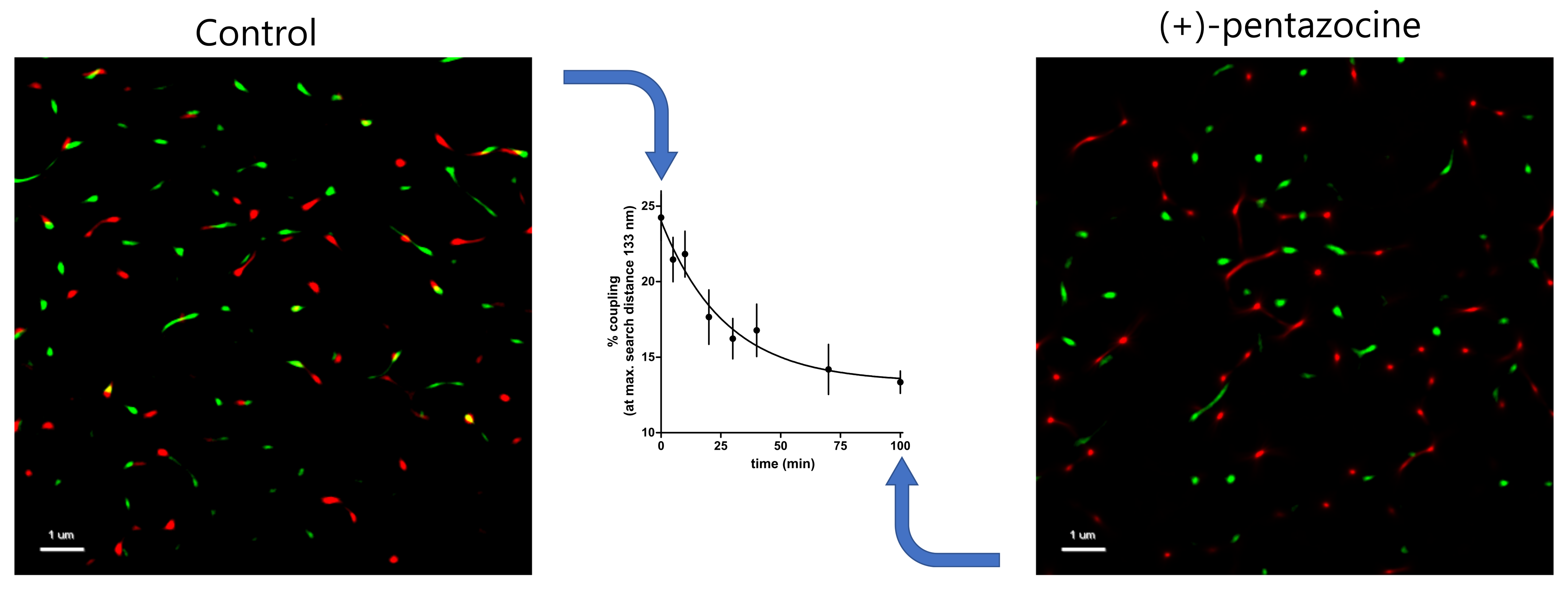

Supplement: S3 Fig — Colocalization demonstrated as spot coupling between Sig1R-YFP (green) and KDEL-mRFP (red) at baseline (Control, left image) and 100 min after activation of Sig1R with 100 nM (+)-pentazocine (right image). Zoomed SRRF images are presented. Multichannel frames were acquired in time-laps mode (100 frames), under HILO illumination with an exposure time of 100 ms and sequential switching between 488 nm and 561 nm lasers with 60× oil (NA 1.49) objective lenses (Olympus Corp., Tokyo, Japan). Scale bar = 1 μm. A time-dependent decrease in spot coupling was observed between Sig1R-YFP and KDEL-mRFP after activation of Sig1R with 100 nM (+)-pentazocine (middle image). The first data point on the x axis (0 min) indicates baseline measurement (Control). Spot coupling was calculated at a search distance of 133 nm, which corresponds to 5 SRRF pixels. Data are from images of at least 8 more cells from at least two independent experiments. (TIF) [file pone.0268563.s003.tif]
