# Supplementary material for: Intracellular dynamics of the Sigma-1 receptor observed with super-resolution imaging microscopy
Source: PLoS One. 2022 May 18;17(5):e0268563. doi: 10.1371/journal.pone.0268563 (PMC9116656; doi:10.1371/journal.pone.0268563)
Supplement: S1 Table — (A) Specific binding of [3H](+)-pentazocine to membranes of SK-OV-3-Sig1R-YFP cells. (B) Displacement of [3H](+)-pentazocine binding to membranes of SK-OV-3-Sig1R-YFP cells by PRE-084. (PDF) [file pone.0268563.s005.pdf]

S5 Table

Intracellular dynamics of the Sigma-1 receptor observed with super-resolution imaging microscopy

Sergei Kopanchuk [1], Edijs Vavers [2,3], Santa Veiksina [1], Kadri Ligi [1], Liga Zvejniece [2], Maija Dambrova [2,3] and Ago Rinken [1*]

[1]University of Tartu, Institute of Chemistry, Tartu, Estonia

[2]Latvian Institute of Organic Synthesis, Riga, Latvia

[3]Riga Stradins University, Riga, Latvia

S5 Table A. Specific binding of [^{3}H](+)-pentazocine to membranes of SK-OV-3-Sig1R-YFP cells. The specific binding was defined as the difference between total and nonspecific binding, measured in the absence and presence of 10 µM haloperidol, respectively. The data are shown as the mean ± SEM from three independent experiments carried out in duplicate.

**[^{3}H](+)-Pentazocine (nM)**	**[^{3}H](+)-Pentazocine bound (pmol/mg protein ± SEM)***
0.03	0.02 ± 0.01
0.2	0.06 ± 0.04
0.3	0.22 ± 0.07
0.6	0.56 ± 0.21
2.2	1.00 ± 0.24
6.8	2.45 ± 0.51
17.0	6.41 ± 0.80
31.8	6.82 ± 1.04

S5 Table B. Displacement of [^{3}H](+)-pentazocine binding to membranes of SK-OV-3-Sig1R-YFP cells by PRE-084. The binding of 2 nM [^{3}H](+)-pentazocine was measured in the presence of indicated concentration of PRE-084 and normalized to [^{3}H](+)-pentazocine binding in the absence of PRE-084 in the particular experiment. The data are shown as the mean ± SEM from four independent experiments carried out in duplicate.

PRE-084 (μM)	log[PRE-084 (M)]	[^{3}H](+)-pentazocine bound (% ± SEM)*
100	-4.0	-1 ± 2
10	-5.0	5 ± 2
1	-6.0	26 ± 6
0.3	-6.5	72 ± 12
0.1	-7.0	80 ± 9
0.01	-8.0	92 ± 11
0.001	-9.0	100 ± 4
0.0001	-10.0	97 ± 7
