# Supplementary material for: Intracellular dynamics of the Sigma-1 receptor observed with super-resolution imaging microscopy
Source: PLoS One. 2022 May 18;17(5):e0268563. doi: 10.1371/journal.pone.0268563 (PMC9116656; doi:10.1371/journal.pone.0268563)
Supplement: Attachment — Submitted filename: Response_to_reviewers_REV1.docx [file pone.0268563.s007.docx]

# Response to Reviewers

PONE-D-21-39296

Dear editor, dear referees,

Thank you very much for the effort, kind words, and useful and insightful suggestions. We have discussed the suggestions with all the authors and formulated following answers and performed described changes in the manuscript. We hope that after these changes and answers, the manuscript correspond to the high standard of the *PLOS One* publication criteria.

Herewith we present point to point answers and explanations of all questions raised by the editor and referees.

## **Reviewer #1:**

Sergei Kopanchuk et al examined intracellular dynamics of the sigma-1 receptor (Sig-1R) by using the super-resolution imaging microscopy.

The Sig-1R has been proposed as a novel therapeutic target of several human diseases such as neurodegenerative disorders, drug abuse, and virus infection. The unique cell biological properties include: (1) Sig-1Rs form clusters at endoplasmic reticulum (ER) subdomains and (2) translocate upon activation by Sig-1R agonists. However, detailed characterization of subcellular localization and dynamics of Sig-1Rs wait further investigations. The technical barriers against the characterization include (1) the transfection technique which is essential for live-cell imaging may alter properties or location of the protein, especially when expressed at the ER membrane; (2) only a small portion of Sig-1Rs translocate upon their activation; (3) since Sig-1Rs move mainly on the ER membrane with a limited distance (not organelle-to-organelle translocation), high resolution is required (higher than that of conventional confocal microscopy).

Kopanchuk et al overcome a few above-mentioned barriers. They used SRRF to detect and analyze the intracellular dynamics of Sig1R. They also employed BacMam technology which was optimized to avoid the generation of protein aggregates and achieve equal expression in the majority of cells

SRRF with SODA experiments not only confirms the previously suggested Sig-1R localization (co-localization with both ER and mitochondoria markers), but also provide convincing data; namely, even though the high-intensity spots of these proteins are very close to each other, they only partially overlap, and locate within the distance of 100 nm. More importantly SRRF enables the quantitative measurement of Sig-1R agonist-induced translocation and average velocity.

The manuscript was well written and provided data should be important for a wide range of scientists (i.e., in the fields of cell biology, molecular biology and pharmacology). The experimental design, used technologies, data analyses, and data interpretation are all reasonable and scientifically sound.

Thank you very much for the nice summary and pointing out the most important points of the manuscript.

Having said this, I would like to provide a few suggestions to increase the quality of the paper.

1. Figure 3 includes the most important data, but its resolution is not high enough (This problem might be caused by its conversion to pdf during the processing of the submission). Please increase the quality of the image.

We would like to thank the Reviewer for noting this issue. The quality of the image was indeed significantly reduced after its conversion to pdf file format. However, during the file submission, we also uploaded tif file of this figure, with dimensions 4500 × 4582 pixels, which must provide a sufficient resolution and quality for the publication. To increase the size of each panel, we decided to split the figure to Fig 3 and Fig 4, in the revised version of the manuscript, and high-resolution versions (4211 × 6341 pixels and 4866 × 1630 pixels) were uploaded with the manuscript to the Editorial Manager.

2. It may be important to provide data that suggests the subcellular Sig-1R-YFP is similar/equal to that of endogenous Sig-1Rs. The subcellular membrane fractionation followed by western blotting should answer to this question.

Thank you for the very important question, which unfortunately cannot be directly answered, as there are very limited possibilities to detect WT Sig-1R.

We, and several our colleagues have encountered several issues with using commercially available Sig1R antibodies due to the unspecific staining. For example, stained Sig1R knockout mice samples are indicated below:

Figure. Representative images, demonstrating unspecific activity of commercially available Sig1R antibodies. For the staining, brain slices (35 µm) from CD-1 background wild-type (WT) and Sig1R knockout (Sig1R-/-) animals were used (unpublished data). Images demonstrate the Sig1R staining intensity in CA1 and DG regions of the mice hippocampus. Staining was performed on frozen, free-floating brain slices using antibodies at a dilution of 1:200 and 1:50 for the Abcam and Santa Cruz antibodies, respectively.

This has been also clearly demonstrated in the paper by Mavlyutov and colleagues (Mavlyutov *et al*., Neuroscience 2016;331:148-57, Figure 2). Our colleagues from several other laboratories have encountered the same problem with other suppliers. Therefore we are very careful with experiments using commercially available Sig1R antibodies. The fact that Sig1R antibodies can give several extra bands in the Western blots with the unknown identity has been mentioned even on the homepage of Abcam antibody (Anti-Sigma1-receptor antibody - C-terminal, ab53852) and is visible even in knockout conditions (Human SIGMAR1 (Sigma1-receptor) knockout HEK-293T cell line (ab266619)).

Due to limitations described above, unfortunately, we are not able to provide reliable comparison of untagged and tagged sigma-1 receptor expression by Western blotting or immunofluorescence as an alternative method.

Therefore, we strongly rely on number of previously published data that confirm that C-terminal tagging of Sig1R does not alter its cellular localization or intracellular function (Sharma *et al*., J Biol Chem 2021;297(5),101299; Zhemkov *et al*., Elife 2021;10:e65192; Yano *et al*., Front Neurosci 2019;13:1356; Hayashi and Su, J Pharmacol Exp Ther 2003;306(2),726-33).

It has been also mentioned that Sig-1Rs form clusters at endoplasmic reticulum (ER) subdomains, and herewith we have shown the localization of Sig1R-YFP together with ER marker KDEL-mRFP (Fig 3) and now also with ER-specific anti-PDI (RL90) - mouse mAb (Fig. S4). Having also in close vicinity markers of mitochondria pmKate2-mito (Fig. 3) and mitochondria-specific anti-Tom20 (D8T4N) - rabbit mAb (Fig. S4), confirming the MAM localization of Sig1R-YFP used in this study.

## **Reviewer #2:**

**Major Comments**

1. The previous authors who verified that Sig1R tagging with YFP did not render it non-functional tested it in NG108-15 cells (Hayashi & Su, J Pharmacol Exp Ther, 2003) and MEF cells (Wong et al., Mol Pharmacol. 2016). Can the authors provide evidence that this same tagging does not alter function in SK-OV-3 cells?

We would like to thank the Reviewer for the comment. We agree that tagging may affect the functions of proteins, and this may substantially vary between cell lines used. Unfortunately, there are no commonly accepted assays for Sig1R to determine its functionality. Here we have used [^3^H](+)-pentazocine binding assay to confirm that the C-terminally tagged protein has retained its ability to bind specific ligands. In addition, we have demonstrated the dynamics of the Sig1R-YFP in SK-OV-3 cells, which is in agreement with earlier findings in NG108-15 cells (Hayashi & Su, J Pharmacol Exp Ther 2003;306(2),726-33; DOI:10.1124/jpet.103.051284).

2. As shown in figure1B, there is little specific binding for endogenous Sig1R by pentazocine. Does this mean the endogenous Sig1R is non-functional? If it is functional, why does the Sig1R-YFP binding show an order of magnitude about 9 times that of the endogenous?

Thank you for the comment. The difference in the binding magnitude seen in Fig. 1B (Fig. 1C in the new version) between naive cells and transfected cells is due to overexpression of Sig1R. Fig. 1C (Fig 1B in the old version) mainly serves as a validation of the expression level of Sig1R reached in SK-OV-3 cells using MultiBacMam expression system. The low level of [^3^H](+)-pentazocine binding in naive cells indicates that the level of endogenous Sig1R is low, and it does not interfere with the effects we can see with Sig1R-YFP. Optimization of transfection conditions revealed that MOI = 3 is sufficient to achieve transfection of the majority of the cells but at the same time is not too high to initiate the generation of protein aggregates into cytoplasmic inclusion bodies. The achieved expression level 11 ± 3 pmol/mg protein [^3^H](+)-pentazocine binding sites does not exceed the level 16.6 ± 3.5 pmol/mg protein determined in rat liver (Hong *et al*., *J Pharmacol Exp Ther*. 2020;373(2):290-301; DOI: 10.1124/jpet.119.262790), indicating that despite the overexpression, the system is close to natural one. On the other side, the binding level of about 1 pmol/mg protein to naive cells does not show that receptors are non-functional. This is the normal level of binding sites, as B_max_ values of [^3^H](+)-pentazocine binding has been found to be 1.73 ± 0.33 pmol/mg protein in MDA-MB-468 cell membranes, and no detectable binding in MCF7 cell membranes (Abbas *et al*., *Frontiers in Pharmacology* 2020;11:309; DOI: 10.3389/fphar.2020.00309). To indicate that we have used relevant expression levels, we added this information also to the discussion part of the manuscript.

3. The authors should re-work Figure2. If these are fixed cells, then it needs to show at least DAPI stained nuclei as well as ER, mitochondria or Golgi stained cells. Provide the merged imaged for these different stains in addition to the Sig1R-YFP fluorescent cells. The current data doesn’t prove that Sig1R-YFP is localised to the ER. It could be simply cytoplasmic localised without preference of the ER. A colocalization analysis is the minimum needed to verify its association with other cellular organelles in this data.

Thank you very much for the very relevant comment. We fully agree that the data in Fig. 2 do not give relevant information about the localization of Sig1R-YFP in cells. In this experimental setup, we have used live cells (not fixed) to indicate that activation of cells with 10 µM PRE-084 does not cause relocalization of the receptors to the basolateral plasma membrane of cells, which can be detected with TIRF microscope. The colocalization of Sig1R-YFP with other cellular components detected in fixed cells are shown otherwhere: with ER on Fig. 3F, with mitochondria Fig. 3E, endosomes Fig, S2A, lysosomes Fig, S2B, caveolae Fig, S2C, peroxisomes Fig, S2D.

As suggested by the Reviewer, we have performed additional immunofluorescence experiments demonstrating the expression of YPF tagged Sig1R in line with markers for ER and mitochondria (S4Fig CF and IL). The obtained staining results are in agreement with the Sig1R-YFP localization demonstrated using MultiBacMam system and expression of organelle-specific fluorescently tagged proteins (Fig. 3 F and E)

4. The authors should provide a Pearson’s correlation co-localisation analysis for Fig S2. This is absolutely essential!

We agree that it is not correct to judge the colocalization of proteins only visually. In the main text, we have used SODA analysis, which is object-based and more relevant in the case of super-resolution imaging. Now we have added the Pearson’s correlation coefficients in Results (lines 403-406) and to the legend of Fig. S2 (lines 776-779)

5. The authors should consider the merits of doing single molecule imaging with Sig1R tagged to wither Halo or mEos. This should be mentioned in the discussion segment.

We would like to thank the Reviewer for the comment. We agree that also other approaches have to be considered in the discussion. Now we have mentioned HaloTag, SNAP-Tags, and mEos4b as possible tools for further studies in the discussion section of the manuscript (lines 498-504).

**Minor Comments**
1. The authors should provide a more detailed data for the expression of Sig1R-YFP than what is shown in Fig.1A. Consider providing confocal images taken on a 63X of 100X objective with some nuclear and ER immunolabelling.

The aim of Fig.1A is an indication of the successful expression of Sig1R-YFP – almost all cells are labeled, and no protein aggregates in cytoplasmic inclusion bodies have formed. More detailed information about the Sig1R-YFP is given in Fig. 2 (scale bar 10 µM) and in Fig. 3 (scale bar 1 µm). As Fig. 1 and 2 are from the live cells, the immunolabelling of organelles would not work for that.

2. The authors should provide a schema of the (pentazocine) binding assay before showing Fig.1B. It is a bit jarring. Also, the authors should provide raw data of the binding assay done on the naïve cells, Sig1R-YFP expressing cells and the PRE-084 treated cells (graphs). Make it a supplementary to Fig.1C & D.

We would like to thank the Reviewer for suggestions A scheme of radioligand binding experiments is now added in Fig. 1A. We have generated S5 Table A and B, including raw data of Fig. 1C & D.

3. The authors should consider using a 100X Obj in TIRF configuration to verify absence of Sig1R-YFP. Also the data will be more convincing if the authors label the cell with Sig1 tagged with multiple tags, for example, GFP and mCherry/mRuby and show by TIRF imaging that these are also not localised to the plasma membrane.

We have used TIRF APON 60× oil objective with numerical aperture (NA) 1.49 and with double tuCam system magnification, which together give 120×. To confirm absence of receptors in the basolateral membrane of cells, the critical parameter is NA, and 1.49 is according to our knowledge highest, which works with ordinary cover glasses.

It has been shown that the addition of a tag to N-terminal, significantly affected the properties of Sig1R (Yano *et al*., Front Neurosci 2019, doi: 10.3389/fnins.2019.01356; Sharma *et al*., J Biol Chem 2021, doi.org/10.1016/j.jbc.2021.101299), while tagging C terminal has little influence. For example, the C-terminal tag of Sig1R with GFP has a similar expression pattern as observed in our study (Zhemkov *et al*., Elife 2021. doi: 10.7554/eLife.65192). To avoid the possibility that effects found are specific only for SK-OV-3 cells, we did similar assay by transfecting of HCC-44 cells (non-small cell lung carcinoma), and the obtained very similar expression pattern of Sig1R-YFP (see representative image below), and no expression/localisation to the plasma membrane could be detected.

Representative image of the expression of Sig1R-YFP (MOI = 3, green) and KDEL-mRFP (red) in the HCC-44 cell line. Scale bar = 10 µm.

4. Are the cells treated with PRE-084 the same as in the control images? If yes, it seems PRE-084 has effects in shrinking the cell.

Thank you for noting this. Yes, cells treated with PRE-084 are the same as in the control images (cells before application of the ligand). The cell shrinking was observed only in live cells (long-term imaging) and was not observed in treated cells which were fixed before the imaging. We believe that the shrinking is caused by the illumination of live cells for imaging.

5. In line 273, the authors should provide a reference where 10 µM of PRE-084 was used. If no reference is available, then the authors should provide a concentration gradient data, for example, 0 -150 uM with time showing the (absence/presence) of effect PRE-084 on Sig1-YFP in these cells.

10 µM PRE-084 has been found to be the optimal concentration in cell experiments. For example, 10 µM PRE-084 had the maximal effect in BiP dissociation assays (Martin *et. al.* (2020). *Epilepsy & Behavior* 105: 106989)., and downregulating ERS-mediated apoptotic genes (Jiang et al. (2020), *Aging*, 12: 9041; DOI: 10.18632/aging.103166). We refer to this now also in the text of the manuscript . (lines 298-299)

6. The authors should re-word lines 290 – 293, such that it ends with Fig S2D) instead of Fig S2C).

Thank you for this comment. We have reworded lines 316-319 and lines 400-402 in the Result part so that figures are mentioned in logical order.

7. The authors should consider placing the colour scheme naming in Fig.3H and not only place this information inside the figure legend.

Thank you for pointing this out! We have reorganized Fig 3 to Fig 3 and Fig 4 and included the color scheme in Fig 4B.
